# Changes in pharyngeal aerobic microflora in oral breathers after palatal rapid expansion

**DOI:** 10.1186/1472-6831-6-2

**Published:** 2006-01-21

**Authors:** Angela Pia Cazzolla, Giuseppina Campisi, Grazia Maria Lacaita, Marco Antonino Cuccia, Antonio Ripa, Nunzio Francesco Testa, Domenico Ciavarella, Lorenzo Lo Muzio

**Affiliations:** 1 Department of Dentistry ad Surgery, University of Bari, Bari, Italy; 2 Department of Dental Sciences "G. Messina", University of Palermo, Palermo, Italy; 3 Department of Surgical Sciences, University of Foggia, Foggia, Italy

## Abstract

**Background:**

The purpose of this study was to investigate in oral breathing children the qualitative and quantitative effects on aerobic and facultatively anaerobic oropharyngeal microflora of respiratory function improved by rapid palatal expansion (RPE).

**Methods:**

In an open clinical trial, we studied 50 oral breathers, aged 8 to 14 years and suffering from both maxillary constriction and posterior cross-bite. At baseline, patients were examined by a single otorhinolaryngologist (ENT), confirming nasal obstruction in all subjects by posterior rhino-manometric test. Patients were evaluated three times by oropharyngeal swabs:1) at baseline (T = 0); 2) after palatal spreading out (T = 1); and 3) at the end of RPE treatment (T = 2). With regard to the microbiological aspect, the most common and potentially pathogenic oral microrganisms (i.e. Streptococcus pyogenes, Diplococcus pneumoniae, Staphylococcus aureus, Haemophilus spp, Branhamella catarrhalis, Klebsiella pneumoniae, Candida albicans) were specifically detected in proper culture plates, isolated colonies were identified by means of biochemical tests and counted by calibrated loop. The data were analyzed by means of the following tests: Chi-square test, Fisher's exact test and Wilcoxon's test.

**Results:**

After the use of RME there was a statistically significant decrease of Staphylococcus aureus stock at CFU/mLat T1(P = 0.0005; Z = -3,455 by Wilcoxon Rank test) and T2 (P < 0.0001; Z = -4,512 by Wilcoxon Rank test) vs T0. No significant changes were found for the other examined microrganisms.

**Conclusion:**

Our data suggest that RPE therapy in oral breathers may strongly reduce the pathogenic aerobic and facultatively anaerobic microflora in the oral pharynx after a normalization of the upper airways function, and may reduce the risk of respiratory infections.

## Background

Aerobic and facultative anaerobic bacteria are prevalent among the bacterial populations of the human body, particularly on mucous membrane surfaces. In the oral cavity these bacteria are found on the tooth surface (above all in subgingival plaque), in the saliva, on the tongue surface and in the tonsillar crypts. [1]

Genera commonly found in the oral cavity are Actinomyces, Arachnia, Bacteroides, Bifidobacterium, Eubacterium, Fusobacterium, Lactobacillus, Leptotrichia, Peptococcus, Streptococcus, Propionibacterium, Selenomonas, Treponema, and Veillonella. [2]

Their role in dental caries, periodontal disease, root canal infections, infections of the hard and soft oral tissue, as well as their importance as foci for disseminated infectious disease is well established. Poor oral hygiene and periodontal disease may promote oropharyngeal colonization by potential respiratory pathogens (Klebsiella pneumoniae, Pseudomonas aeruginosa, Staphylococcus aureus, etc.). [3] Furthermore, aerobes and facultative anaerobes are the most numerous components of the normal human oropharyngeal bacterial flora, and they are therefore a common cause of bacterial infections of the upper respiratory tract which have an endogenous origin (bronchitis, sinusitis, otitis, pneumonia) [4]; in addition to bacteria, fungi, mainly represented by *Candida albicans*, may colonise the oral ecosystem as well as protozoan. (Table 1)

**Table 1:** Bacteria commonly found on the surfaces of the pharynx

*Staphylococcus epidermidis*	++	Streptococcus pyogenes*	+	*Mycoplasmas*	+
Staphylococcus aureus*	+	*Neisseria sp*.	++	*Mycobacteria*	+/-
*Streptococcus mitis*	+	*Klebsiella pneumoniae*	+/-	*Lactobacillus sp*.	+
*Streptococcus salivarius*	++	Neisseria meningitidis*	++	*Hemophilus influenzae*	+
Streptococcus mutans *	+	*Corynebacteria*	+	*Pseudomonas aeruginosa*	+/-
Enterococcus faecalis*	+/-	*Actinomycetes*	+	*Proteus*	+
*Streptococcus pneumoniae*	+	*Spirochetes*	+	Enterobacteriaceae (Escherichia coli)*	+/-

++ = nearly 100% + = common +/- = rare * = potential pathogen.

Asterisks indicate members of the normal flora that may be considered major pathogens of humans.

In detail, *Staphylococci *spp are Gram+ bacteria aerobic and facultative anaerobic, round and with a diameter of 0,8-1; they are normally located at every site of the human body (skin, conjunctiva, nose, pharynx, mouth, lower intestine, anterior urethra, vagina). The most important species is *S. aureus*, responsible for soft tissue pathologies like abscess, stomatitis, angular cheilitis, maxillary osteomyelitis, mastoiditis and parotitis [5]. The enzymes produced by *S. aureus *and released outside the cell are several, some in direct relation to its pathogenic activity (a-, β- and t-emolisin, coagulase, hyaluronidase, exfoliatin, leucocidin, lipase [6], and staphylokinase). Also, *Streptococci *spp are Gram+ bacteria aerobic and facultative anaerobic, capsulate and stationary; they are round, from 1 to 15 μ in diameter and disposed in couples or a chain. Different streptococcal species are present on skin and mucous surfaces (mouth, pharynx, intestine, trachea, vagina), constituting the majority of the oropharyngeal microbial population. Most of them may cause several diseases especially local septic lesions (e.g. tonsillitis, otitis, dental abscess, caries, pharyngitis), flogistic processes in a cavity (e.g. meningitis, pleurisy, pneumonia), systemic diseases (e.g. septicaemia, rheumatic fever) or a fibrin vegetation in a cardiac valve [7]. *Neisserie *and *Branhamella *spp are Gram- cocci, they may be aerobe or anaerobe, stationary, asporogenous and often with a capsule. *N. meningitidis *represents actually a pathogenic species for human meningeal suppurating infections [8].

*Branhamella catarrhalis*, an inhabitant of the upper respiratory tract, is an important cause of a pathological process (bronchitis, maxillary sinusitis, meningitis, endocarditis, otitis and pneumonia) [10].

*Haemophilus influenzae *is one the most important and frequent etiologic agent of meningeal infections, laryngitis, endocarditis or respiratory infections in children [9].

*Klebsiella pneumoniae *is present in the pharynx of only 1–6% of sound people. Causes a serious, often fatal, pneumonia principally in debilitated patients [11].

In this complex microbiological scenario, the respiratory function has got a notable influence not only on the oral habitat but also on the development of the stomatognatic system; indeed, correct respiratory dynamics, through the nasal cavity, are a fundamental stimulus for its anatomical development. Especially in childhood, human beings may breath orally with several immediate and long-standing consequences. The anatomical picture in an oral breather, with a contraction of transversal diameters of the upper maxilla (ogival palatal shape and bilateral cross-bite) is the consequence of an increased resistance of the nasal ways to the flow of air.

For this kind of orthodontic problem, the primary therapeutic approach is the use of a rapid palatal expansor (RPE), which determines a sudden non-surgical detachment of the palatal bones by use of force (> 500 gr), over a short period of time. The rapid palatal expansor was firstly invented and described by E.H. Angell in 1860; however this technique only became widespread in the 70s'. The primary aim of this device is to provide a lateral expansion force of such an intensity as to verify the movement of the basal maxillary bones at the suture level (median palatal, maxillary-zygomatic and pterigo-maxillary sutures) instead of a simple re-modelling of the alveolar arch. At the synfibrosis stage the bony surfaces will rapidly detach and this space will be occupied by fibrous connective tissue which will soon change into a bony structure.

The application of such an apparatus determines the following results:

• an enlargement of the upper maxillary osseous base and its equilibrium with the mandible base;

• certain degree of nasal septum deviation correction

• significant reduction in nasal resistance [12]

• an improvement of respiratory functions [13]

Several researchers observed significant changes in the microflora of the nasopharynx in children subjected to adenoidectomy for chronically hypertrophied and infected adenoids [14,15], but nobody investigated a correlation between RPE, reduction in nasal resistance and pharyngeal microflora.

The aim of the present study was to verify if RPE therapy is able to determine changes in the oral microflora among oral breathing children.

## Methods

### Sample

In the present open clinical trial, the study group was recruited in secondary hospital base and composed of 50 children (range 8–14 years), consecutively admitted for orthodontic treatment (i.e. RPE). Inclusion criteria were European ethnic origin and mouth breathing reported by parents and clinically observed by a dentist specialist in Orthodontics. A child has been considered as an oral breathers if his/her mouth is open at rest, tension of peri-oral muscles is visible when the mouth is closed, he/she complains about nasal obstruction and more often breathes through the mouth [16]. Exclusion criteria were nasal allergic conditions or airway obstruction due to adenoids and previous orthodontic treatment. Subjects did not undergo any type of antibiotic treatment or pharmacological therapy during the observation period which lasted 6 months. The expansion regimen was of 3 turns/day (1 turn = 0.2 mm), until the required expansion was achieved. After the end of the ERP successful active phase, the appliance was left in place for 6 months. Even if the controlled clinical trials represent the ideal study design, we decided against this approach on ethical grounds in view of the severe clinical condition of the patient.

### Clinical recordings

The first step was that all patients were examined by a single ENT. Nasal obstruction was confirmed by posterior rhynomanometric test, performed at ENT Clinic (University of Bari, Bari, Italy) using Athos 300 rhino-manometer, according to the protocol recommended by the Manufacturer.

Three consecutive pharyngeal swabs were carried out: the 1^st ^before application of RPE (T = 0), the 2^nd ^after palatal spreading out (T = 1), and the 3^rd ^at the end of RPE treatment (T = 2). Microbiologically, we looked for the following microrganisms: *Streptococcus pyogenes, Diplococcus pneumoniae, Staphylococcus aureus, Haemophilus spp, Branhamella catarrhalis, Klebsiella pneumoniae, Candida albicans*.

It is a question of microrganisms that may be considered major pathogens of humans.

Each microbiologic sample carried out in the posterior pharynx and palatal tonsillar was repeated three times with different sterile swabs. Two swabs were directly used for seeding in culture plates, the third was saved with 500 μl of sterile saline and underwent vortex for 1 min. The obtained suspension was used for further seeding with a 10 μl of seed medium. Specifically, to detect Streptococci, 5 ml of Streptococcus broth were added to the residual saline; this broth is useful for Streptococcus isolation and their culture was subsequently performed on blood Agar (5% lamb red blood cells which inhibit *Haemophilus haemoliticus*), by adding Optochine which is an inhibitor of *Diploccocus pneumoniae *and Bacitracine which inhibits *Streptococcus pyogenes*. Further species were identified by means of biochemical tests.

In order to detect the *Staphylococcus aureus *a Chapman broth was used (75% NaCl, mannitole, phenole red); colonies found as positive for pathogens underwent a coagulase test. In order to search for *Neisseria *and *Hemophilus *spp: agar-blood incubated at 5–10% CO_2 _and agar-chocolate incubated at 35° were respectively used. To detect Enterobacteria, Agar Mac-ConcKey (biliary salts, red neutral lactose) was used; for mycetes: Agar- Sabouraud (dextrose, peptone) with cloramphenicole and gentamicine as bactericides was used. Identification of *Candida albicans*, the only fungal species observed, was made after incubation in blood serum at 37°C for 2–3 hours; the comparison between germinative tubules and pseudo-hyphae, the observation of Albicans ID2 containing chromogenic exosamine were also carried out. After the surface sowing on plate for spatulation and opportune incubation, we proceeded to enumeration of the positive cultures for the species searched and count of the number of colonies which developed for single plate (vital count, count in plate or count of the colonies), which is correlated to the explored anatomic zone numerousness. It has been indicated the number of colonies growing on each plate and expressed by Colony Forming Unit (CFU/ml). Two count methods can be distinguished:

-direct method (it uses the corpuscles count chamber and it counts both alive cells and dead cells);

-indirect method or of the vital count or count in plate or count of the colonies, sow and subsequent counting of the developed colonies (sensible method). We count only the alive cells which are able to proliferate and form colonies on suitable agaric land. The microbiological research of *Streptococcus pyogenes*, showed only the presence or not of microrganisms. After the end of RPE treatment, no posterior rhino-manometric test was performed again, since this datum did not constitute a major end-point of the trial.

### Statistical analysis

Data were analyzed by means of the computer packages S-Plus 4.0 (Cambridge, UK). The Chi-square test was used to assess statistical differences among categorical variables; Fisher's exact test was used when the frequency observed was less than 5; in all the evaluations p-values = 0.05 were considered statistically significant. Furthermore, in order to evaluate significance of differences (expressed in CFU/ml) at T = 0, T = 1 and T = 2 the Wilcoxon's test was used.

## Results

Microbiological findings are described in Table 2. In detail, *Streptococcus pyogenes *was present in 5 patients (10%) at T0, in the same 5 patients at T1, and then disappeared at T2, although the difference did not reach statistical significance (P = 0,07). Neisseria meningitis, Diplococcus pneumoniae and Haemophilus spp were not found in none of the 50 patients at T0, T1 and T2. A significant difference was found for *Klebsiella pneumoniae *and *Branhamella catarrhalis*, both present in 5 patients at T0 (10%) and in no patient at T1 and T2 (P = 0,006). *Candida albicans *was found in 15 patients (30%) at T0, in 19 (38%) at T1 and in 10 (20%) at T2, without any significant change (P = 0,141), the same for *Staphylococcus aureus- *stock b, which was found in 8 patients (16%) at To and at T1, and in 2 patients (4%) at T2 (P = 0,103). A significant variation was found in the three therapy steps for *Staphylococcus aureus *– stock a present in 37 patients (74%) at T0 in 34 (68%) at T1 and in 22 (44%) at T2(P 0,005).

**Table 2:** Species and frequency rates at T0, T1 and T2 in a sample investigated (*n *= 50)

**Species**	**T0**	**T1**	**T2**	**X^2^**	P*
*Candida albicans*	30% (15/50)	38% (19/50)	20% (10/50)	3,924	0,141
*Staphylococcus aureus- stock a*	74% (37/50)	68% (34/50)	44% (22/50)	10,696	0.005*
*Staphylococcus aureus- stock b*	16% (8/50)	16% (8/50)	4% (2/50)	4,545	0,103
*Branhamella catarrhalis*	10% (5/50)	0	0	10,345	0,006*
*Neisseria meningitidis*	0	0	0	-	-
*Klebsiella pneumoniae*	10% (5/50)	0	0	10,345	0,006*
*Haemophilus*	0	0	0	-	-
*Diplococcus pneumoniae*	0	0	0	-	-
*Streptococcus pyogenes*	10% (5/50)	10% (5/50)	0	5,357	0,069

*by means of Chi-Square test

The involvement of a greater number of patients from these last three microbic species allowed the use of the Wilcoxon Signed Rank test in order to evaluate differences expressed in CFU/ml (see Table 3). It was evaluated significant the decrease caused by RPE treatment at T0 vs T1 (Z -3,455; P = 0.0005) and at T0 vs T2 (Z -4,512, P = 0,0001) for *Staphylococcus aureus*-stock a and at T0 vs T1 for *Candida albicans *(Z -3,267; P = 0,0011). There are no significant changes at T0, T1 and T2 for *Staphylococcus aureus- *stock b and for *Candida albicans *at T0 vs T2.

**Table 3:** Means ± DS of the CFU/mL of the main species detected at T0, T1 and T2, respectively.

Species*	T0	T1	Z	P**	T0	T2	Z	P**
Staph.aureus stock a	28,23 (± 53,96)	5,48 (± 5,10)	-3,455	0,0005	28,23 (± 53,96)	3,07 (± 6,08)	-4,512	0,0001
Staph.aureus stock b	5,23 (± 9,13)	3,46 (± 3,30)	-0,105	0,916	5,23 (± 9,13)	2 (± 4,88)	-2,521	0,011
Candida	5,95 (± 5,20)	1,8 (± 1,00)	-3,267	0,0011	5,95 (± 5,20)	2,25 (± 3,04)	-1,792	0,07

*For *Branhamella catarrhalis, Neisseria meningitidis, Klebsiella pneumoniae, Haemophilus, Diplococcus pneumoniae *and *Streptococcus pyogenes*

CFU/mL values were found in little number of patients and quantity, not adequate for statistical analysis.

**by means of Wilcoxon Signed Rank Test.

## Discussion

The orthodontic relevance of nasal obstruction and its presumed effect on facial growth and head posture continues to be debated. The growth of the maxilla depends on the hormone activity, passive activities (i.e. sutures) as well as active dynamics. The lateral expansion of the maxilla is directly conditioned by the development of the nasal capsula and pterigoid floors and indirectly by the expansion of the eyeballs [17]., KIThe main characteristics of nasal obstruction are mouth breathing, open bite, cross-bite, narrow external nostrils [18]. Other features include an increase in lower facial height, incompetent lip posture, right angle of the mandibular plane and V-shaped maxillary arch [19]. The clinical extra-oral picture in these patients is conspicuous and is labelled as 'adenoidal faces'.

Oral respiration disrupts those muscle forces exerted by tongue, cheeks and lips upon the maxillary arch. Intra-orally, the dentist might expect to find a narrow maxillary arch with a high palatal vault, a posterior cross-bite, a class II or III dental malocclusion, open-bite.

RME is the primary device used in the orthodontic treatment of insufficient transverse dimension of the maxillary base [20]. The treatment with RME determines an improvement of respiratory function [21]. A large number of bacterial species heavily colonize the upper respiratory tract (nasopharynx). The predominant species are non-hemolytic and alpha-hemolytic streptococci and neisseria, but sometimes pathogens such as *Streptococcus pneumoniae*, *Streptococcus pyogenes*, *Haemophilus influenzae*, *Neisseria meningitides *and *Staphylococcus aureus *colonize the pharynx. A reduction in ventilation through the nasal cavity may allow a permanence of mucous secretions, which modify microbial growth and leads to upper respiratory tract infection. The literature indicates that the species responsible for this latter condition are the following: *Staphylococcus aureus, Streptococcus pyogenes, Streptococcus Enterobactery, Streptococcusα-haemolytic, Neisseriaceae, Staphylococcus aureus, Pneumococcus, Streptococcus β-haemolytic group A, Streptococcus β-haemolytic non A, Pseudomonas aeruginosa, Haemophilus influenzae, Branhamella catarrhalis *[14].

The purpose of this study was to verify if rapid palatal expansion was able to determine quantitative and qualitative changes in anaerobic and facultative anaerobic pharyngeal micro-flora in young oral breathers, an issue not properly estimated in so far.

Our main finding is that pathogenic micro-flora of the oral pharynx may be reduced after the use of RPE in oral breathers. In particular, we observed in breathers a notable presence, at baseline, of *Staphylococcus aureus*, an important pathogen, and its reduction after RME. *Candida albicans *showed a particular pattern with an initial reduction (T0 *vs *T1) with a successive re-colonization at T2 in the same patients, likely due to a persistence of different predisposal factors [22,23] or, in some cases, colonized some of them *ex novo*. Although in a little sample size and without controls, the findings of this study suggest that the improvement of nasopharyngeal airway adequacy RME-related is associated to a decreased presence of *Staphylococcus aureus-*stock a. The long-term benefits of the RPE- therapy in terms of respiratory infections was not investigated. Further studies with larger numbers of patients, presence of a control group and more details of the clinical status regarding the frequency of infections, may be required for further evaluation of this interesting issue.

## Pre-publication history

The pre-publication history for this paper can be accessed here:


